# The Transcriptional Regulator Lrp Contributes to Toxin Expression, Sporulation, and Swimming Motility in *Clostridium difficile*

**DOI:** 10.3389/fcimb.2019.00356

**Published:** 2019-10-17

**Authors:** Kuan-Yu Chen, Jagat Rathod, Yi-Ching Chiu, Jenn-Wei Chen, Pei-Jane Tsai, I-Hsiu Huang

**Affiliations:** ^1^Department of Microbiology and Immunology, College of Medicine, National Cheng Kung University, Tainan, Taiwan; ^2^Department of Earth Sciences, National Cheng Kung University, Tainan, Taiwan; ^3^Center of Infectious Disease and Signaling Research, National Cheng Kung University, Tainan, Taiwan; ^4^Department of Medical Laboratory Science and Biotechnology, College of Medicine, National Cheng Kung University, Tainan, Taiwan

**Keywords:** *Clostridium difficile*, leucine-responsive regulatory protein, transcriptional regulator, toxin, sporulation, motility

## Abstract

*Clostridium difficile* is a Gram-positive, spore-forming bacterium, and major cause of nosocomial diarrhea. Related studies have identified numerous factors that influence virulence traits such as the production of the two primary toxins, toxin A (TcdA) and toxin B (TcdB), as well as sporulation, motility, and biofilm formation. However, multiple putative transcriptional regulators are reportedly encoded in the genome, and additional factors are likely involved in virulence regulation. Although the leucine-responsive regulatory protein (Lrp) has been studied extensively in Gram-negative bacteria, little is known about its function in Gram-positive bacteria, although homologs have been identified in the genome. This study revealed that disruption of the lone *lrp* homolog in *C. difficile* decelerated growth under nutrient-limiting conditions, increased TcdA and TcdB production. Lrp was also found to negatively regulate sporulation while positively regulate swimming motility in strain R20291, but not in strain 630. The *C. difficile* Lrp appeared to function through transcriptional repression or activation. In addition, the *lrp* mutant was relatively virulent in a mouse model of infection. The results of this study collectively demonstrated that Lrp has broad regulatory function in *C. difficile* toxin expression, sporulation, motility, and pathogenesis.

## Introduction

Leucine-responsive regulatory protein (Lrp) is a global transcriptional regulator involved in modulating various metabolic functions and physiology and is widely distributed among prokaryotes and archaea (Newman and Lin, 1995; Brinkman et al., 2003; Peeters and Charlier, 2010). The *Escherichia coli* Lrp is the most researched regulator of the Lrp family and is estimated to directly or indirectly control the gene expression of approximately one third of all *E. coli* genomes (Kroner et al., 2019). In *E. coli*, Lrp monitors a general nutritional state by sensing the concentrations of leucine and alanine in the cell and regulating genes involved in entering the stationary phase of growth (Bouvier et al., 1998; Ihara et al., 2017). The binding of the amino acid effector by Lrp can promote or reduce the effector's regulatory effects through transcriptional activation or repression. However, in some cases, regulation by Lrp is leucine independent (Newman et al., 1992; Brinkman et al., 2003). Although many *lrp* homologs have been identified through genome analysis and multiple paralogs are present within the genome in some cases, only a handful have been studied in detail, and thus the functions of most homologs remain unclear. In addition to its role in bacterial growth in nutrient-limited environments, Lrp acts as a virulence regulator in numerous including *Salmonella enterica* serovar Typhimurium (Baek et al., 2009), *V. cholera* (Lin et al., 2007), *Xenorhabdus nematophila* (Richards and Goodrich-Blair, 2009), *Mycobacteria* (Deng et al., 2011), and *Proteus mirabilis* (Fraser and Hughes, 1999).

*Clostridium difficile* is a spore-forming, anaerobic Gram-positive toxin producer transmitted among humans through the fecal–oral route and causing antibiotic-associated diarrhea worldwide (Leffler and Lamont, 2015). Because of high morbidity, mortality (Dembek et al., 2018), and relapse (Hota and Poutanen, 2018) rates, *C. difficile* infection (CDI) constitutes a major threat to global health care and is accountable for a substantial financial burden (Nanwa et al., 2015) [estimated as ~€3 billion per annum in the European Union and US$4.8 billion in the United States Dembek et al., 2018].

Multiple studies have focused on the virulence determinants of *C. difficile* in *ex vivo* and *in vitro* experiments and have provided a comprehensive overview on virulence and pathogenicity. Toxin A (TcdA) and toxin B (TcdB) are major secretory toxins that are responsible for the massive fluid secretion, colonic tissue necrosis, and inflammation associated with CDIs (Farrow et al., 2013; Leffler and Lamont, 2015). A third toxin, namely cytolethal distending toxin (CDT), is a binary toxin that act as auxiliaries to exotoxins during severe pathogenicity (Janoir, 2016). Furthermore, the ability to form stress-resistant spores, flagella, Type IV pili, and numerous other surface adhesive proteins enhances the colonization efficiency and virulence of *C. difficile* (Abt et al., 2016). Related studies have identified numerous regulators of the aforementioned virulence factors; however, a comprehensive picture of virulence gene regulation in *C. difficile* remains to be formed (Smits et al., 2016).

Although the role of Lrp as a global regulator in Gram-negative bacteria is widely known, little is known about its role in Gram-positive bacteria, even though homologs have been identified from genomes. In Gram-positive bacteria, another regulator, CodY, may have a partially analogous effect on Lrp (Sonenshein, 2005). CodY plays a global Lrp-like role in *Bacillus subtilis* and its relatives by regulating the anabolic, catabolic, differentiation, and virulence pathways (Levdikov et al., 2017). *B. subtilis*-encoded Lrp-like protein (LrpC) was shown to play a role in the growth phase transition (Beloin et al., 1997) and the transport of branched-chain amino acids (Belitsky et al., 1997). Therefore, the role of Lrp or Lrp-like proteins in most Gram-positive bacteria remains ambiguous. Genome analysis of *C. difficile* strains 630 and R20291 has revealed a single gene annotated as *lrp*; however, this genetic determinant has yet to be researched further. Hence, the present study aimed to understand the role of Lrp in *C. difficile* gene regulation.

## Materials and Methods

### Bacterial Strains, Plasmids, and Growth Conditions

Details of *C. difficile* strains and plasmids are provided in Table 1. Strains were grown and maintained at 37°C in a Don Whitley DG250 anaerobic workstation under anaerobic conditions (10% H_2_, 10% CO_2_, 80% N_2_; Don Whitley Scientific Ltd., Bingley, United Kingdom). *C. difficile* strains were routinely cultured in modified brain heart infusion-supplemented (BHIS) medium, 70:30 sporulation medium (Childress et al., 2016), or chemically defined minimal medium (CDMM) (Karasawa et al., 1995). For solid media, agar was added to a final concentration of 1.5%. All media were supplemented with 15 μg/ml thiamphenicol, 40 μg/ml lincomycin, 5 μg/ml erythromycin, and 300 μg/ml cycloserine when necessary. *E. coli* strains were grown in L-broth or on L-agar as described in a previous report (Donachie and Begg, 1970), and plasmids were maintained by 30 μg/ml chloramphenicol. All antibiotics were purchased from Sigma-Aldrich (St. Louis, MO, USA).

**Table 1:** Strains and plasmids used in this study.

**Strain**	**Genotype/Description**	**Origin**
***E. coli***		
DH5α	F^−^ϕ80lacZΔM15 Δ(lacZYA-argF) U169 recA1 endA1 hsdR17(rk^−^,mk^+^) phoA supE44 thi-1 gyrA96 relA1 λ^−^	Invitrogen
CA434	HB101 carrying the Incβ conjugative plasmid R702	(Williams et al., 1990)
***C. difficile***		
R20291	Clinical isolate	Gift from Dr. Daniel Paredes-Sabja
630	Sequenced referenced strain	Gift from Dr. Daniel Paredes-Sabja
630Δerm	Erm^S^ derivative of strain 630	Gift from Dr. Daniel Paredes-Sabja
JC01	R20291 *lrp::erm*. Insertional *lrp* mutant	This study
JC02	630Δerm *lrp::erm*. Insertional *lrp* mutant	This study
JC03	R20291 *lrp*::*erm* pYC03	This study
JC04	630Δerm *lrp*::erm pYC04	This study
**Plasmids**		
pMTL007C-E5	Derived from pMTL5402F by inserting the group II intron, ErmBtdRam2, and ltrA ORF from, Tm^R^ and Erm^R^	(Heap et al., 2007)
pYC01	pMTL007C-E5 with group II intron targeted to *lrp* (CD630_35440)	This study
pYC02	pMTL007C-E5 with group II intron targeted to *lrp* (CDR20291_3379)	This study
pMTL83151	*E. coli*/*C. difficile* shuttle vector	(Heap et al., 2007)
pYC03	pMTL83151 containing *lrp* coding region and 500-bp promoter region of CD630_35430	This study
pYC04	pMTL83151 containing *lrp* coding region and 500-bp promoter region of CDR20291_3378	This study

### Genetic Manipulation

The *lrp* mutant was generated in *C. difficile* R20291 and 630Δerm by using the ClosTron method described in a previous report (Heap et al., 2010). In brief, the L1.LtrB intron present in plasmid pMTL007C-E5 was retargeted to CD3379 (strain R20291) and CD3544 (strain 630) by using intro-retargeting primers (Table 2). Plasmid retargeting was performed as described in a previous report (Kuehne et al., 2010). The resultant plasmid, pYC01, was transferred to *C. difficile* R20291 and 630Δerm through conjugation, as described in a previous report (Bouillaut et al., 2011). Thiamphenicol-resistant transconjugants were plated on BHIS agar plates containing lincomycin (20 μg /ml; *C. difficile* R20291) or erythromycin (5 μg/ml; *C. difficile* 630) for the selection of potential mutants. Putative mutants were then screened through polymerase chain reaction (PCR) with primers Screen-F/R and Erm-F/R (Table 2). To complement the *lrp* mutant strain, the *lrp* coding sequence was fused to the upstream promoter to exclude the intervening open reading frame predicted immediately upstream of *lrp* through PCR with primers Lrp promoter-F/R and Lrp-F/Lrp-SalI-R (Table 2). The two PCR fragments were fused through overlap extension PCR with primers Lrp promoter-F/Lrp-SalI-R. The resultant PCR fragments digested by BamHI and SalI were cloned into pMTL84151 to generate plasmids pYC02 and pYC03, which were then introduced through conjugation into the 630 *lrp* mutant and R20291 *lrp* mutant with *E. coli* CA434 acting as a donor. A list of all plasmids and strains constructed in this work is presented in Table 1.

**Table 2:** Oligonucleotides used in this study.

**Primer name**	**Sequence (5′-3′)**	**Description**
*lrp*-63-IBS primer	AAAAAAGCTTATAATTATCCTTAATTTCCATGAAGGTGCGCCCAGATAGGGTG	Intron-retargeting primer
*lrp*-63-EBS1d primer	CAGATTGTACAAATGTGGTGATAACAGATAAGTCATGAAGGATAACTTACCTTT CTTTGT	Intron-retargeting primer
*lrp*-63-EBS2 primer	TGAACGCAAGTTTCTAATTTCGGTTGAAATCCGATAGAGGAAAGTGTCT	Intron-retargeting primer
EBS universal primer	CGAAATTAGAAACTTGCGTTCAGTAAAC	Intron-retargeting primer
Screen-F	ATGGATTTACAGATTACAGAATC	Lrp specific primer
Screen-R	CGTTGATAGTATAACAGAGGTCT	Lrp specific primer
Erm-F	ACGCGTTATATTGATAAAAATAATAATAGTGGG	Erm marker specific primer
Erm-R	ACGCGTGCGACTCATAGAATTATTTCCTCCCG	Erm marker specific primer
Lrp promoter-BamHI-F	AATUGGATCCLINECAAAGTTTGAAGCTCAC	Complementation of *lrp* mutant
Lrp promoter-R	GTAACATCCATTATTTCTCTCCTT	Complementation of *lrp* mutant
Lrp-F	ATGGATGTTACAGATTACAGAATC	Complementation of *lrp* mutant
Lrp-SalI-R	ATAUGTCGACLINEATTAAGGATACTTAATGGTC	Complementation of *lrp* mutant
**qRT-PCR primers**		
qRpoC-F	CTAGCTGCTCCTATGTCTCACATC	Reference gene
qRpoC (DPS630)-R	CCAGTCTCTCCTGGATCAACTA	Reference gene
qRpoC (R20291)-R	CCAGTTTCACCTGGATCAACTA	Reference gene
qLrp-F	GGTTTAACTTCTCCTGCAGTTTC	*lrp*
qLrp-R	CTCTGCCTAATGAATCTGGGTT	*lrp*
qTcdA-F	AAAGCTTTCGCTTTAGGCAGTG	*tcdA*
qTcdA-R	CTCTATGGCTGGGTTAAGGTGTTG	*tcdA*
qTcdB-F	GATCACTTCTTTTCAGCACCATCA	*tcdB*
qTcdB-R	AGCTTCTTAAACCTGGTGTCCATC	*tcdB*
qTcdR-F	CATTATGAAGAGGGAGAAACAGATTT	*tcdR*
qTcdR-R	CTAGACAACTCAAAAGTCTTATTCAG	*tcdR*
qTcdC(DPS630)-F	GAGCACAAAGGGTATTGCTCTA	Strain 630 *tcdC*
qTcdC(DPS630)-R	AAATGACCTCCTCATGGTCTTC	Strain 630 *tcdC*
qDtxA(R20291)-F	GAAGACCATGAGGAGGTCATTT	R20291 *tcdC*
qDtxA(R20291)-R	CATGGTTCAGCATCAGACAATTT	R20292 *tcdC*
qFliC-F	GGGAAGAAACGTAAATGCACAA	*fliC*
qFliC-R	GCATCATCAGCAGCTCTCTTA	*fliC*
qCcpA-F	AATCCACCTGCTAGAAGCTTAGT	*ccpA*
qCcpA-R	AGCAACCTCTTCTATCCCATTT	*ccpa*
qCodY-F	AGGAAGCGGTCAAAGATTAGG	*codY*
qCodY-R	ACAGTTGCACTGTATTCAGCTA	*codY*
qSpo0A-F	AGCGCAATAAATCTAGGAGCA	*spo0A*
qSpo0A-R	TGGCTCAACTTGTGTAACTCTAT	*spo0A*
qSigE-F	TGACTTTACACTTTCATCTGTTTCTAGC	*sigE*
qSigE-R	GGGCAAATATACTTCCTCCTCCAT	*sigE*
qSigF-F	CGCTCCTAACTAGACCTAAATTGC	*sigF*
qSigF-R	GGAAGTAACTGTTGCCAGAGAAGA	*sigF*
qSigG-F	CAAACTGTTGTCTGGCTTCTTC	*sigG*
qSigG-R	GTGGTGTTAATACATCAGAACTTCC	sigG
qCD1579-F	AGTAAGGGTATGGGCAAAGTATTACA	CD1579/CD1476
qCD1579-R	CCACTTCATTTGAGAACAACTCTTTG	CD1579/CD1476
qSigD-F	GAATATGCCTCTTGTAAAGAGTATAGCA	*sigD*
qSigD-R	TGCATCAATCAATCCAATGACTCC	*sigD*

### Bacterial RNA Extraction and Real-Time Quantitative Reverse Transcription PCR

Overnight culture of *C. difficile* strains was refreshed in Trypticase Yeast extract medium (TY) (for toxin-associated genes) or 70:30 medium (for sporulation-associated genes) and grown anaerobically at 37°C. At designated time points, bacterial cells were harvested through centrifugation, and the total RNA was isolated using RNAprotect Bacteria Reagent (Qiagen, Venlo, Netherlands) in accordance with the manufacturer's instructions. Genomic DNA was removed using RQ1 RNase-free DNase (Promega, Madison, WI, USA). RNA was reverse transcribed into complementary DNA by using SuperScript™ II Reverse Transcriptase (Invitrogen, Carlsbad, CA, USA) and random primers (Thermo Fisher Scientific, Waltham, MA, USA) in accordance with the manufacturers' instructions. The relative transcriptional level of genes of interest in the tested strains were measured with real-time quantitative reverse transcription PCR (qRT-PCR) by using the 2x qPCRBIO SyGreen Mix Hi-Rox (PCR Biosystems, London, United Kingdom) and gene-specific primers (Table 2) in accordance with the manufacturer's instructions. The StepOnePlus™ Real-Time PCR System (Applied Biosystems, Foster City, CA, USA) was also employed. Data were analyzed using the 2-ΔΔCt method with normalization to the *rpoC* reference gene and stated reference condition. At least three independent samples were analyzed. Statistical analyses were conducted using GraphPad Prism 6.0. (GraphPad Software, San Diego, CA, USA).

### TcdA and TcdB Western Blotting

*C. difficile* strains were grown overnight in TY medium with or without Tm and then diluted 1:50 in fresh medium and let grown for an additional 14 h. The supernatant was collected through centrifugation at 4,000 rpm for 10 min and filtered with a 0.22-μm sterile syringe filter. Proteins were extracted using trichloroacetic acid (Sigma-Aldrich) and acetone, and normalize in equal concentration aliquots as described in a previous report (Schwarz et al., 2007). Protein concentration in cell-free supernatant was quantified using standard Bradford protein estimation. Dried pellets were dissolved in sample dye and subjected to sodium dodecyl sulfate–polyacrylamide gel electrophoresis and Western blotting. Anti-TcdA and -TcdB antibodies (R&D Systems Inc., Minneapolis, MN, USA) were added at 1,000-fold dilution, and goat anti-mouse IgG conjugated to Horseradish Peroxidase (HRP) (Thermo Fisher Scientific) was added at 10,000-fold dilution. HRP activity was detected using the BioSpectrum^®^ Imaging System™ (Analytik Jena US LLC, Upland, CA, USA) and ECL Select™ Western Blotting Detection Reagent (GE Healthcare, Chicago, IL, USA). Csp1 (CD2831) was used as internal loading control (Hensbergen et al., 2015). Each Western blot also included 4 μl of a BLUeye Prestained Protein Ladder (GeneDireX).

### Cell Culture and Cytotoxicity Assay

Caco-2 and Vero cells were cultured in Dulbecco's modified Eagle medium containing 10% fetal bovine serum in a humidified incubator with 5% CO_2_ and maintained at 37°C. Cell viability was determined through detachment with 1,000 U/ml trypsin and 0.5 mM ethylenediaminetetraacetic acid and counted using a LUNA-FL Dual Fluorescence Cell Counter (Logos Biosystems, Gyeonggi-do, Korea). Subsequently, the cells were seeded into 96-well tissue culture test plates (SPL Life Sciences Co., Ltd., Gyeonggi-do, Korea) at a density of 5 × 10^4^ cells per well and incubated overnight at 37°C in an atmosphere containing 5% CO_2_. For the cytotoxicity experiments, *C. difficile* strains were cultured in TY medium for 24 h. Bacteria cultures were centrifuged at 4°C and 4,000 rpm for 10 min, and supernatants were collected. After filter sterilization, the supernatants were diluted serially in 2-fold and then incubated with cells for 24 h. The cytotoxic titers were expressed as the highest dilution exhibiting a >50% cytopathic effect (Rosenbusch et al., 2012). The samples were measured in triplicate, and statistical analyses were conducted using GraphPad Prism 6.0.

### Sporulation Efficiency Assays

*C. difficile* strains were inoculated into BHIS medium supplemented with 0.l% taurocholate (Sigma-Aldrich) and grown to the mid-log phase. The cultures were subsequently diluted 100 fold in 70:30 sporulation medium (Childress et al., 2016). All cultures were incubated anaerobically at 37°C and monitored for growth and spore production. At designated time points, concentrated culture suspensions were placed on a thin 0.5% agarose pad applied to a slide and imaged with a 100X oil immersion objective by using an Olympus CX31 Upright Microscope (Olympus Life Science). Three fields of view were acquired for each strain by using a Tucsen ISH500 complementary metal–oxide–semiconductor camera (Tucsen Photonics, Fuzhou, China). A minimum of 1,000 cells from each strain were used to calculate the percentage of spores (the number of spores divided by the total imaged population) (Burns et al., 2011). Statistical analyses were conducted using GraphPad Prism 6.0.

### Swimming Motility Assay

*C. difficile* strains were grown overnight in BHIS medium supplemented with 0.1% taurocholate (Sigma-Aldrich), diluted 100 fold in fresh BHIS medium, and grown to the mid-log phase. To measure swimming motility, agar tubes containing BHIS medium (0.175% agar) were stab inoculated and grown anaerobically at 37°C overnight. To highlight the degree of motility, black and white images of tubes were captured, and areas of growth were determined in triplicate (Aubry et al., 2012; Gro et al., 2018).

### Biofilm Formation Assay

An overnight culture of *C. difficile* strains was refreshed to the late exponential to early stationary phase (OD600 = ~0.8) in BHIS broth and then diluted 100-fold in fresh medium (BHIS + 0.1 M glucose) on 24-well polystyrene plates. The plates were then incubated anaerobically at 37°C for 72 h. To quantify the biofilm mass, supernatants were carefully decanted, two times washed by PBS and retaining biofilms were allowed to dry at room temperature for 30 min. Two percent crystal violet was added to each well for 30 min and then removed through methanol treatment for an additional 30 min. Extracted dye contents were quantified by measuring the absorbance at 595 nm with a Multiskan™ GO Microplate Spectrophotometer (Thermo Fisher Scientific) (Purcell et al., 2017). At least three independent samples were analyzed. Statistical analyses were conducted using GraphPad Prism 6.0.

### Animal Virulence Studies

Specific-pathogen-free 8-weeks old male C57BL/6 mice were housed in the Laboratory Animal Center of National Cheng Kung University (NCKU). All mice were maintained and handled in accordance with the guidelines of the Institutional Animal Care and Use Committee (IACUC) of NCKU. Moreover, all animal studies were performed following a protocol approved by the IACUC of NCKU (approval no. NCKU-IACUC-102-149) and the NCKU Biosafety and Radiation Safety Management Division. The *C. difficile* animal infection model was performed as described in previous reports (Hung et al., 2015). To condition the mice for CDI, they were fed drinking water containing an antibiotic mixture of 0.4 mg/mL vancomycin, 0.215 mg/mL metronidazole, 0.4 mg/mL kanamycin, 0.035 mg/mL gentamycin, and 850 U/mL colistin for 5 days before infection. All antibiotics were purchased from Sigma-Aldrich. Vancomycin and metronidazole were omitted to avoid disrupting *C. difficile* colonization on the day before infection. Esomeprazole dissolved in phosphate-buffered saline (PBS) was administered to all mice via oral gavage 12 h prior to infection (18.55 mg/kg) and immediately before infection (4.82 mg/kg). One day before infection, all of the mice received clindamycin (4 mg/kg) intraperitoneally. On the day of infection, all of the mice were challenged via oral gavage an overnight culture of *C. difficile* strain R20291 wild type and *lrp* mutant adjusted to 1 × 10^8^ CFU/mL. Two days after infection, all the mice were euthanized by CO_2_ asphyxia. Histopathological analysis was conducted to evaluate mucosal damage and inflammation. Resected colon tissue samples were fixed in 4% formaldehyde buffered with PBS and then embedded in paraffin. Sections were stained through hematoxylin and eosin or periodic acid-Schiff (PAS) staining.

## Results

### *lrp* Gene Arrangement and Translated Protein Sequence Analysis

Genome analysis of prokaryotes has revealed that members of the Lrp/AsnC family of transcriptional regulators are widely distributed in most eubacteria and archaea (Brinkman et al., 2003). Sequence analysis using SyntTax (Oberto, 2013) revealed that genomes of *C. difficile* strains 630, R20291, and CD196 contain one copy of the *lrp*/AsnC gene. The synteny of the neighboring *lrp* gene in *C. difficile* strains was found to be conserved, and the location of *lrp* was revealed to be identical in all sequenced *C. difficile* strains. Further, a comparison of previously reported *C. difficile* genomes showed 100% identity in Lrp amino acid sequences among strains CD196, R20291, 630, 630Δerm, and R1 (Supplementary Figure 1). This observation suggests that *lrp* is not recently acquired and likely serves a common function in all *C. difficile* strains.

**Figure 1:**
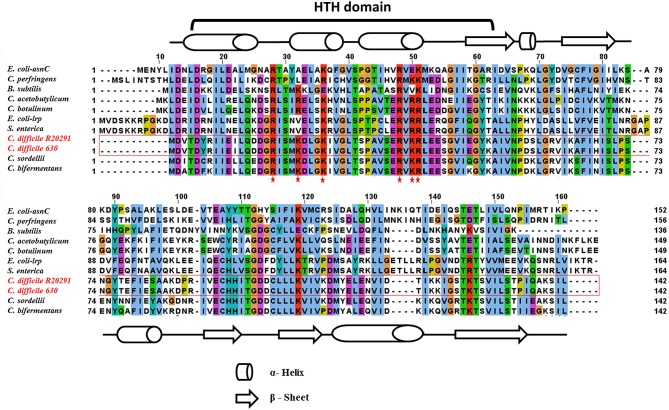
Amino acid sequence alignment of *C. difficile*–encoded Lrp with other members of the Lrp/AsnC family. *C. difficile* Lrp amino acid sequences shared on average about 40% identity with the other Lrp sequences. The residues predicted to encode the DNA-binding HTH motif are identified. Secondary structural elements are indicated by barrels (α-helix) and arrows (β-sheet). Conserved lysin and arginine residues within the HTH domain are highlighted with a red star.

In this study, the *C. difficile–*encoded Lrp/AsnC protein amino acid sequence was compared with a set of eubacterial Lrp representatives. In Gram-negative bacteria, the most well-characterized member of the Lrp/AsnC family is *E. coli lrp* (Tani et al., 2002), whereas in Gram-positive bacteria, it is *B. subtilis* LrpC (Beloin et al., 1997). The result of amino acid sequence alignment of Lrp in *E. coli, B. subtilis, Salmonella*, and *C. difficile* is shown in Figure 1. The amino acid sequence of *C. difficile* Lrp was 40 and 32% identical with *E. coli* Lrp and *B. subtilis* LrpC, respectively. A PROSITE pattern search identified a putative helix-turn-helix (HTH) motif at the N-terminal of *C. difficile* Lrp (Kroner et al., 2019). In addition, the C-terminal of *C. difficile* Lrp was predicted to contain a βαββαβ-fold (αβ-sandwich) that is also found in *E. coli* Lrp (Brinkman et al., 2003). Furthermore, *C. difficile* Lrp contains a conserved lysine residue located within the HTH domain previously identified to be required for the DNA-binding ability (Qin et al., 2016).

### Generation of *lrp* Mutant in *C. difficile* Strains R20291 and 630Δerm

**Figure 2:**
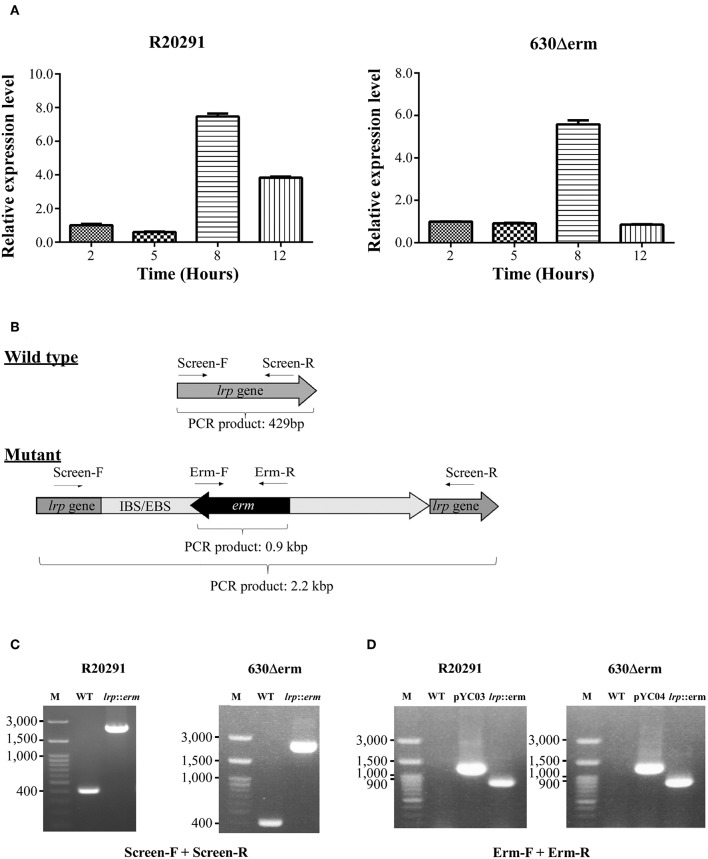
Growth phase–dependent *lrp* transcriptional profiling and insertional inactivation of *lrp* in *C. difficile* R20291 and 630Δerm. **(A)** *lrp* transcript level at different time interval representing various growth phases in BHIS were studied: 2 h (lag phase), 5 h (early log phase), 8 h (mid-log phase), and 12 h (late log/early stationary phase). **(B)** An illustration of ClosTron-dependent insertional mutation and primers used. The ClosTron delivery system is encoded on plasmid and consists of a group II intron with an internal retro-transposition-activated marker conferring erythromycin resistance. The group II intron is re-targeted to the desired target gene by altering the sequence of the intron-binding site/exon-binding site region using overlapping PCR. This results in the splicing of the group II intron into the target gene. The locations of primers used to screen for potential mutant are indicated. **(C)** PCR confirmation using primers Lrp-screen-F and Lrp-screen-R. **(D)** Insertion confirmation using primers Erm-F and Erm-R. M, DNA ladder; WT, wild type; pYC03, R20291 *lrp* ClosTron plasmid; pYC04, 630Δerm *lrp* ClosTron plasmid; *lrp, erm*: *lrp* mutant.

To determine the growth-phase-specific *lrp* expression in *C. difficile* strains 630 and R20291, cells grown in BHIS were harvested at different growth incubation time at 2, 5, 8, and 12 h. The 2-h lag phase was considered basal expression, and relative expression was analyzed, showing 5.4-fold-higher (*p* < 0.0001) and 7.4-fold-higher (*p* < 0.0001) expression at the log phase time point (8 h) in strains 630 and R20291, respectively (Figure 2A).

To address the function of Lrp in *C. difficile*, we utilized the insertional mutation system known as ClosTron, which is enabled by a group II intron from *Lactococcus lactis* (Kuehne and Minton, 2012). The general gene knockout process is represented schematically in Figure 2B and described in detail in the Materials and Methods section. The *lrp* knockout was first screened using primers that targeted the entire *lrp* ORF, which generated a 429 bp product in the wild type while the same primer set amplified a PCR product of ~2.2 kbp in the mutant through intron insertion (Figure 2C). Furthermore, the presence of the erythromycin resistance cassette in the genome was confirmed by PCR (Figure 2D). As expected, using the ClosTron mutator plasmid as a template generated a product of ~1,300 bp; by contrast, no product was observed when wild type genomic DNA was used as a template (Figure 2D). The same primer set amplified a 900-bp PCR product in the *lrp* mutant, thereby suggesting successful incorporation of the intron into the genome. In addition, the relatively small size indicated that the Td2 intron had been excised from the erythromycin resistance marker due to intron insertion.

### Nutrient-Specific Effect of *lrp* Mutation on the Growth of *C. difficile*

**Figure 3:**
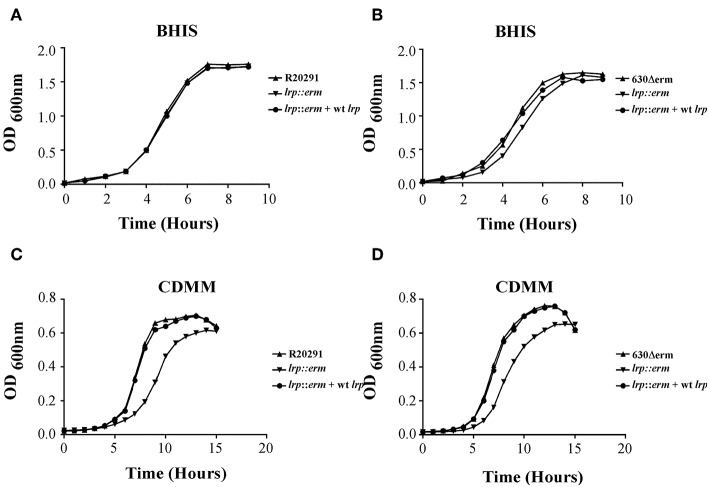
Growth kinetics of *C. difficile* R20291 and 630Δerm *lrp* strains. 12-h cultures of the parental strains, *lrp* mutant strains, and complemented strains were used for inoculation into fresh BHIS (nutrient rich) **(A,B)** and CDMM (nutrient poor) **(C,D)** medium. The OD600 values were recorded at fixed time points. Data are represented as the mean ± standard error of the mean, and the results are representative of at least three independent experiments.

Previously, Lrp was shown to be required for optimal growth under nutrient limiting stress conditions (Kaiser and Heinrichs, 2018). To determine whether *C. difficile* Lrp is involved in growth regulatory processes, growth analysis was conducted for strains R20291 and 630Δerm and their respective *lrp* mutants, as well as complemented strains. Growth was analyzed in two different nutrient availabilities: BHIS broth (nutrient rich) and CDMM (nutrient limiting). No significant growth differences were observed between the parental strains, *lrp* mutants, and complemented strains in R20291 and 630Δerm grown in BHIS (Figures 3A,B). On the contrary, *lrp* mutant strains exhibited delayed growth in CDMM, resulting in lower optical density up to early stationary phase (Figures 3C,D). The *lrp* mutant in strain R20291 was determined to have a doubling time of 103.43 min while the parental strain and the complemented strain had a doubling time of 69.30 and 71.44 min, respectively. A similar growth defect was observed in the 630Δerm *lrp* mutant compared to the parental strain (67.94 min compared to 58.24 min; Figure 3D). The growth defect was restored in *lrp* mutants transformed with wild type *lrp*-expressing plasmid, thereby demonstrating that the growth defect had been caused by the inactivation of the *lrp* gene.

### Lrp Is a Repressor of Toxin A and B Expression

**Figure 4:**
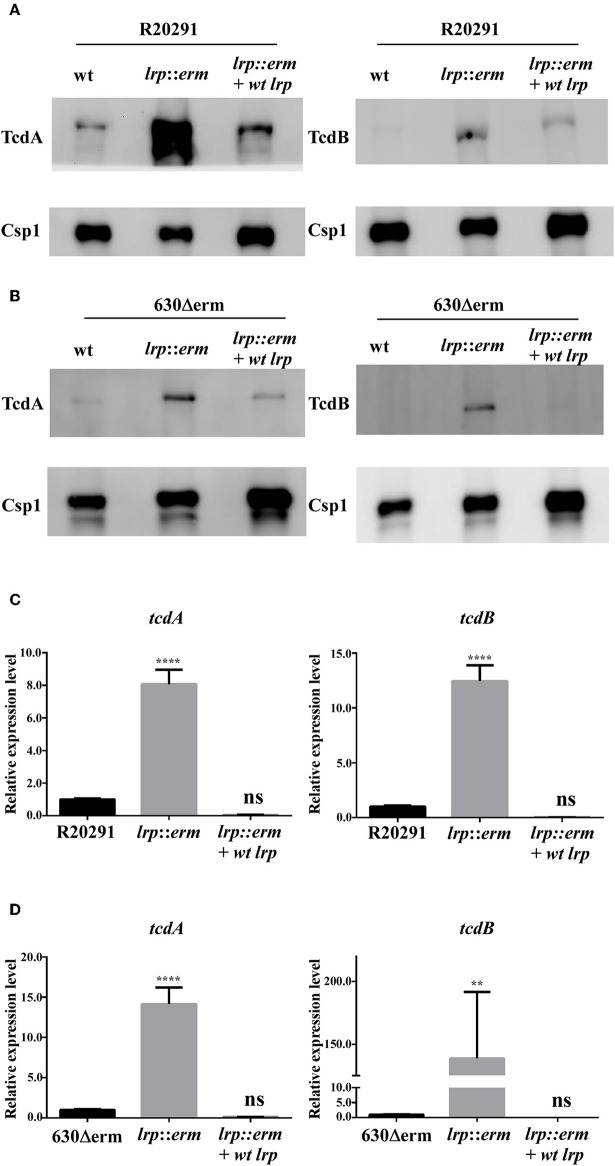
Lrp is a repressor of toxin A and B production in *C. difficile*. Supernatants collected from an overnight culture (15 h) grown in TY broth were used to determine toxin A and B production. **(A)** and **(B)** Western blot of *C. difficile* toxin A (TcdA) and toxin B (TcdB). The TcdA (308 kDa) and TcdB (270 kDa) protein levels were higher in the *lrp* mutant in both R20291 and 630Δerm. Anti-Csp1 served as a loading control [Csp1, cell surface protein (CD2831)]. **(C,D)** Quantitative Real Time PCR (RT-qPCR) analysis to assess *tcdA* and *tcdB* expression in the parental, *lrp* mutant, and complemented strains of R20291 and 630Δerm. mRNA expression levels were measured using culture grown to the mid-exponential stationary growth phase (8-h growth time point); 16s ribosomal RNA was used for reference. Data are represented as the mean ± standard error of the mean, and the results are representative of at least three independent experiments (ns, non-significant; ^**^p < 0.01; ^****^p < 0.0001).

Toxin production in *C. difficile* can be regulated by multiple regulatory circuits (Martin-Verstraete et al., 2016). To understand the potential role of Lrp in toxin production, supernatants from the parental strain, *lrp* mutant, and complemented strain were analyzed for toxin A (TcdA) and toxin B (TcdB) production. As shown in Figure 4A, the levels of TcdA and TcdB from the *lrp* mutant were significantly higher than those from wild type R20291. The increase in toxin production observed in the mutant was reversed in the complemented strain (Figure 4A). Similar results were observed when toxin A and B were detected in strain 630Δerm; the inactivation of *lrp* resulted in increased toxin production and this increase was abolished when the mutant was complemented (Figure 4B). As a loading control, all samples were also subjected to detection with an antibody specific to Csp1 (CD2831), a known protein secreted by *C. difficile* (Hensbergen et al., 2015).

We hypothesized that the increases in toxin A and B production observed in the *lrp* mutant were regulated transcriptionally. Therefore, we further investigated the gene expression levels of these toxin genes. The *lrp* mutant showed a significant increase in toxin gene expression in both tested strains. The inactivation of *lrp* resulted in 14-fold (*p* < 0.0001) and 8-fold (*p* < 0.0001) increases in *tcdA* expression in strains 630Δerm and R20291, respectively (Figures 4C,D). Furthermore, transcription of *tcdB* increased 12-fold (*p* < 0.0001) in strain R20291 (Figure 4C) and 138-fold (*p* < 0.01) in strain 630Δerm (Figure 4D). Complementation of the *lrp* mutant in both strains repressed the expression of both *tcdA* and *tcdB* to levels similar to those observed in the wild type strains.

**Figure 5:**
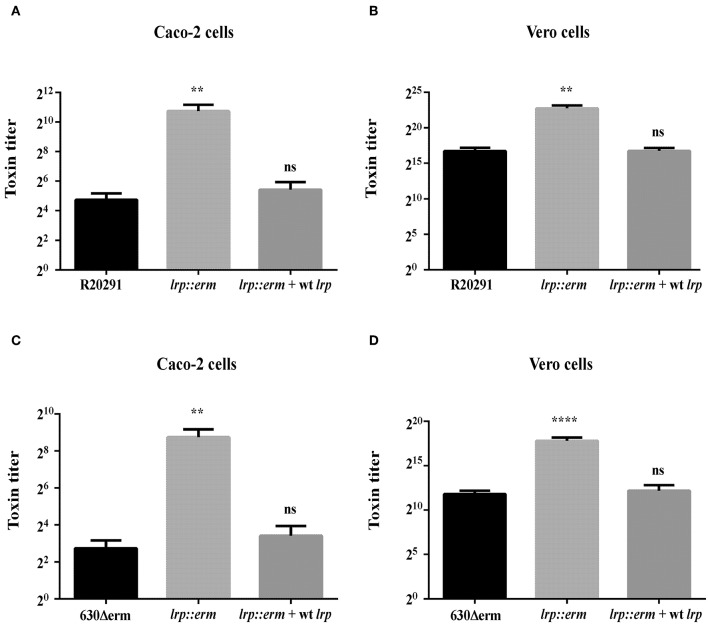
Inactivation of lrp increased the cytotoxic effects of R20291 and 6301erm. Supernatants of R20291 and derivatives **(A,B)** and 630(Δ)erm and derivatives **(C,D)** from overnight cultured in TY medium were added to cultured cells and cytotoxicity was detected after 24 h. Caco-2 cells were used to determine the cytotoxicity of toxin A, while Vero cells were used to determine the cytotoxicity of toxin B. Statistical significance was set as follows: ^**^p < 0.01; ^****^p < 0.0001). Data are represented as the mean ± standard error of the mean of three independent experiments.

To confirm that the increased TcdA and TcdB production in the *lrp* mutant strain corresponded to increased cytotoxicity against mammalian cells, we incubated Caco-2 and Vero cells with filtered supernatants from overnight cultures of the wild type, *lrp* mutant, and complemented strains. As shown in Figure 5, the supernatants from R20291 *lrp* mutants displayed higher cytotoxicity toward both Caco-2 and Vero cells while the effect was restored in the complemented strain. Similar effects were also observed for the 630Δerm *lrp* mutant strain and complemented strain (Figures 5C,D). Collectively, these results demonstrated that Lrp is a repressor of toxin A and B production.

### Lrp Affects the Transcription of Multiple Transcriptional Regulators

**Figure 6:**
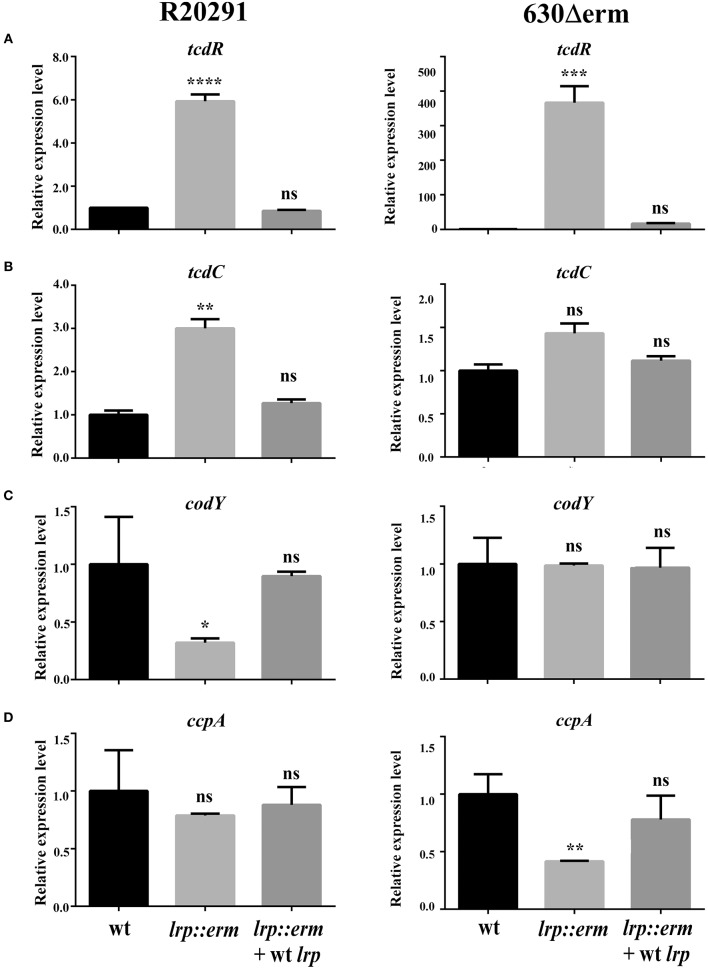
Effect of lrp inactivation on the transcriptional status of potential toxin regulators. Quantitative Real Time PCR (qRT-PCR) was conducted to assess the expression levels of known and putative toxin regulators. mRNA expression levels were measured using culture grown to the mid-exponential stationary growth phase (8-h growth time point); 16s ribosomal RNA was used for reference. All experiments were carried out in triplicate (*N* = 3). Data were analyzed by one-way analysis of variance and Dunnett's multiple-comparison test (ns, not significant; ^*^p < 0.05; ^**^p < 0.01; ^***^p < 0.001; ^****^p < 0.0001). **(A)** tcdD transcription. **(B)** dtxA/tcdC transcription. **(C)** codY transcription. **(D)** ccpA transcription.

To underpin the role of Lrp in regulating toxin production in *C. difficile*, the transcriptional levels of various known and extensively studied toxin regulators were investigated using RT-qPCR. The transcriptional level of TcdR—a known repressor of both *tcdA* and *tcdB*—was significantly upregulated in the *lrp* mutant. The *tcdR* gene in strain R20291 showed 6-fold (*p* < 0.0001) increase. In stark contrast to its parental strains, strain 630Δerm showed 366-fold (*p* < 0.001) a substantial increase in *tcdR* expression compared to respective parental strains (Figure 6A). In the complemented strains of both R20291 and 630Δerm, increases in transcriptional levels were restored. The transcriptional level of *tcdC* (R20291), a putative toxin repressor, was slightly upregulated in the *lrp* mutant (3-fold, *p* < 0.01) in the *lrp* mutant, whereas the effect was restored in the complemented strain. Notably, no significant difference was observed for *tcdC* (homolog) between strain 630Δerm and its *lrp* mutant (Figure 6B).

In addition to TcdR and TcdC, toxin A and B have also been found to be regulated by numerous regulators at the transcriptional level (Martin-Verstraete et al., 2016). CodY is a recognized toxin and nutritional regulator of *C. difficile* (Dineen et al., 2007, 2010). Strain-specific *codY* gene expression profiles were obtained and revealed that the *lrp* mutant of R20291 exhibited an ~3-fold significant reduction in gene expression (*p* < 0.05; Figure 6C). In addition, no significant difference was observed between the 630Δerm parental strain and its *lrp* mutant (Figure 6C). In both *lrp* mutants, complementation with wild type *lrp* resulted in a *codY* gene expression level similar to those of the parental strains.

The catabolite control protein (CcpA) is a pleiotropic regulator that plays a key role in the global transcriptional response to the availability of carbohydrates (Abt et al., 2016). No significant differences in *ccpA* expression were observed between the R20291 parental strain and its *lrp* mutant (Figure 6D). However, inactivation of *lrp* in strain 630Δerm resulted in a 2.3-fold (*p* < 0.01) decrease in *ccpA* expression compared with the parental strain (Figure 6D). The reduction in *ccpA* expression in the 630Δerm *lrp* mutant strain was restored in the complemented strain. In summary, Lrp is a repressor of both TcdA and TcdB in strain R20291 as well as 630Δerm. This repressive effect is likely due to the downregulation of multiple toxin regulators in a strain-specific manner.

### Role of Lrp in *C. difficile* Sporulation

**Figure 7:**
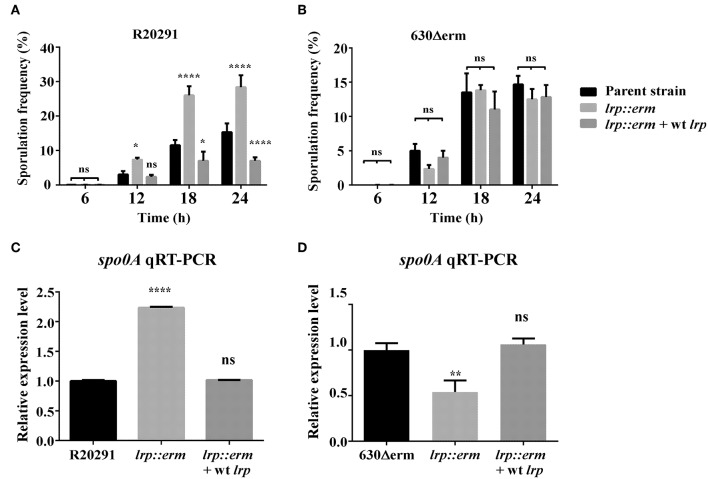
Lrp is a repressor of sporulation in strain R20291. The sporulation frequency were measured by phase-contrast micrograph between parental strain, *lrp* mutant and its complement for strain R20291 **(A)** and 630Δerm **(B)** grown in SMC medium at various time points. **(C,D)** qRT-PCR analysis of spo0A expression for the parental, *lrp* mutant, and complemented strains; 16s ribosomal RNA was used for reference. Data were analyzed by one-way analysis of variance and Dunnett's multiple comparison test. All data are represented as the mean ± standard error of the mean, and the results are representative of at least three independent experiments (ns, not significant; ^*^p < 0.05; ^**^p < 0.01; ^****^p < 0.001).

*C. difficile* sporulation is an important event in defining its virulence and pathogenicity (Abt et al., 2016). To determine Lrp involvement in the bacterial physiology related to spore formation in *C. difficile*, various time course sporulation assay was performed. All the test strains were inoculated into 70:30 sporulation medium, and at various time points, samples were withdrawn and analyzed microscopically to assess the sporulation frequency. The *lrp* mutant of strain R20291 displayed a statistically significant higher sporulation frequency starting at 12 h, and this trend continued until 24 h (Figure 7A). This increase in sporulation efficiency was restored when the mutant was complemented with wild type *lrp*. Notably, this effect of Lrp on sporulation appeared strain dependent as no such difference was observed in strain 630Δerm (Figure 7B). The sporulation efficiency of the *lrp* mutant appeared to decrease, but this change was not statistically significant. To further analyze the role of *lrp* on sporulation, the transcriptional level of *spo0A*, the master regulator of sporulation initiation, was measured (Figures 7C,D). The gene expression of *spo0A* in the R20291 *lrp* mutant strain was found to be significantly increased by 2.2-fold compared with the parental strain (*p* < 0.05; Figure 7C), interestingly, the *lrp* mutant of strain 630Δerm demonstrated 1.9 fold reduction in *spo*0A compared with the corresponding parental strain (Figure 7D).

The sporulation signaling cascade requires the coordinated activation of multiple sigma factors (Paredes-Sabja et al., 2014). To further investigate the role of *lrp* on sporulation, the transcriptional levels of *sigE, sigF, sigG*, and the putative sporulation-associated histidine kinase CD1476 (strain R20291)/CD1579 (strain 630Δerm) were measured (Dineen et al., 2010; Girinathan et al., 2017) (Supplementary Figure 2). The R20291 *lrp* mutant strain showed an increased transcription levels in all three of the tested sigma factors: *sig*E (5.0-fold, *p* < 0.05); *sig*F (2.0-fold, *p* < 0.05); *sig*G (3.0-fold, *p* < 0.05). These results were consistent with the observed increase in *spo0A* expression. By contrast, no differences were observed in the transcriptional level of putative histidine kinase CD1476. Complementation with wild type *lrp* restored the transcription of all three sigma factors to levels similar to that of R20291 (wild type). Notably, the 630Δerm *lrp* mutant strain exhibited decreases in the transcriptional levels of *sig*E (2.9-fold, *p* < 0.05) and *sig*F (1.5-fold, *p* < 0.05), whereas no such difference was observed in *sigG* and CD1579. No significant differences in gene expression were observed when the 630Δerm *lrp* mutant strain was complemented. These results collectively demonstrated that *lrp* affects sporulation in a strain-specific manner both as a repressor of sporulation in R20291 and a possible activator of sporulation in 630Δerm.

### Role of Lrp in Motility and Biofilm Formation

In *C. difficile*, flagella-mediated swimming motility and biofilm formation play key roles in host colonization. To determine whether Lrp affects swimming motility in *C. difficile*, we performed a stabbed soft agar diffusion assay. The *fliC* mutant strain served as a negative control, and the parental strains were considered positive controls. Compared to the parental strain, the R20291 *lrp* mutant strain displayed a defect of swimming motility similar to the *fliC* mutant strain as indicated by the lack of growth dispersion from the central stab line (Supplementary Figure 3A). By contrast, swimming motility was unaffected by the inactivation of *lrp* in strain 630Δerm (Supplementary Figure 3B). Further analysis revealed a significant decrease in the transcriptional level of the flagellar regulator SigD in the R20291 *lrp* mutant compared to the parental strain (Supplementary Figure 4). No significant differences were observed in the transcriptional level of SigD in the 630Δerm *lrp* mutant when compared with the parental strain. Biofilm formation in multi-well plates was measured using crystal violet staining. However, no differences were observed among parental strains, *lrp* mutant, and their complemented strains (Supplementary Figure 4).

### Lrp Is Involved in *C. difficile* Pathogenesis *in vivo*

**Figure 8:**
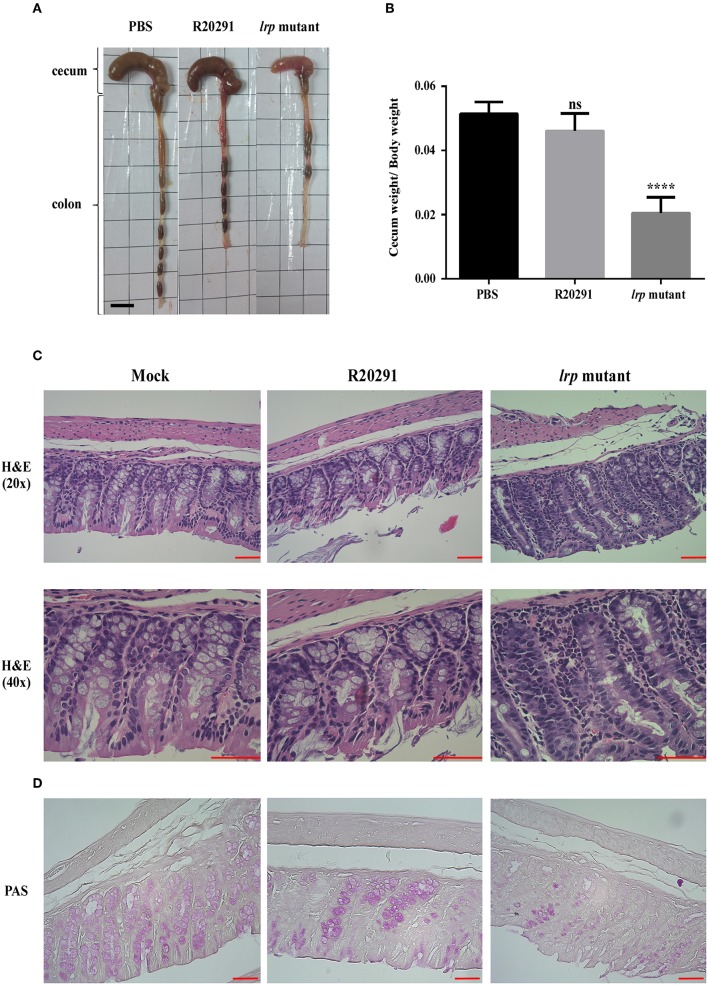
Inactivation of *lrp* resulted in increased inflammation *in vivo*. **(A)** Representative gross views of the cecum and colon from uninfected mice or mice infected with different strains of *C. difficile*. **(B)** Quantification of normalized reduction in cecum weight as an indication of cecum inflammation. **(C)** Representative colon sections stained with hematoxylin and eosin. **(D)** Periodic Acid-Schiff stain. (Magnification × 40 and × 20; scale bar, 5 μm for dimensional comparison between images. The results were analyzed by one-way analysis of variance and Dunnett's multiple—comparison test (ns, not significant; ^****^p < 0.0001).

Finally, to investigate the role of *lrp* in *C. difficile* pathogenesis *in vivo*, we utilized the established mouse model of infection. Mice were infected with either wild type R20291 or its *lrp* mutant. We hypothesized that because of its relatively high toxin production ability, the *lrp* mutant would induce relatively severe inflammation and diarrhea. To effectively observe any potential differences between R20291 and the *lrp* mutant, we used a sublethal dose of *C. difficile* for infection. No significant differences were observed between the PBS control group and the wild type R20291 group in terms of gross cecum, colon morphology as well as cecum weight after infection (Figures 8A,B). Gross views of the colon and cecum indicated greater severity of colitis in the *lrp* mutant group than in the wild type group. In addition, significantly decreased cecum weight was observed in the *lrp* mutant group compared with the wild type group. Histological examination of colon tissue samples revealed that compared with the PBS-treated mice, the R20291-infected mice exhibited an increase of inflammatory cells and greater desquamation of necrotic epithelial cells in their colon mucosa (Figure 8C). Moreover, compared with the R20291-infected mice, the *lrp* mutant–infected mice exhibited a further decrease in crypts, goblet cell depletion, and denser infiltration of inflammatory cells in the colon mucosa. Similarly, compared with the mock mice, the R20291-infected mice revealed depletion of PAS-positive goblet cells in the colon mucosa; this condition was more severe in the *lrp* mutant–infected mice (Figure 8D). The differences in disease severity observed between the R20291-infected and *lrp* mutant–infected groups were not due to colonization rates (data not shown). Overall, these observations suggested that Lrp is involved in the virulence of *C. difficile in vivo*.

## Discussion

Lrp is a highly conserved transcriptional regulator that regulates a wide range of gene expression and pathogenesis in various Gram-negative bacteria, including *E. coli* (Kroner et al., 2019) and *Salmonella* (McFarland et al., 2008). As Gram-negative bacteria exit the exponential growth phase and enter into stationary phase, the *lrp* concentration in cells is elevated (Kaiser and Heinrichs, 2018). Classically, Lrp is traditionally considered to mediate transitions between feast and famine through its reciprocal regulation of amino acid metabolism, wherein biosynthetic genes are activated and catabolic genes are repressed (Tani et al., 2002). Similarly, CodY, a conserved transcriptional regulator in low-GC Gram-positive bacteria (*Firmicutes*)—have similar functionality in sensing the metabolic status of cells to promote adaptation to nutrient limitations (Sonenshein, 2005). In addition to metabolic and physiological genes (amino acid and purine biosynthesis, sugar and amino acid transport, the Krebs cycle, and sporulation and biofilm formation in some species), CodY also regulates virulence gene expression in Gram-positive pathogens [*Bacillus anthracis* (Van Schaik et al., 2009), *C. difficile* (Daou et al., 2019), *Clostridium perfringens* (Li et al., 2013), *Listeria monocytogenes* (Lobel et al., 2015), and others Kaiser and Heinrichs, 2018]. However, although the global role of CodY as a regulator of metabolism and virulence in Gram-positive bacteria has been studied extensively, the global or local regulatory role of Lrp in Gram-positive pathogens remains unknown. Various Gram-positive bacteria possess a conserved copy of *lrp* in their genome (Figure 1). However, the non-pathogenic *B. subtilis* is the only Gram-positive bacteria in which functional characterization of Lrp has been explored (Thaw et al., 2006). Thus, the primary focus of the present study was to elucidate the role of Lrp in the physiology and pathogenesis of pathogenic, Gram-positive *C. difficile*.

The multiple sequence alignment of various Lrp amino acid sequences highlighted conserved residues and functional features. A PROSITE pattern search on *C. difficile* Lrp identified a putative HTH motif at the N-terminal, as marked in Figure 1. *E. coli lrp* HTH domain shared 44.4% sequence identity with the *C. difficile* putative HTH domain. Various DNA- and protein-binding amino acid residues were identified using the REPROFSec online tool (https://ppopen.rostlab.org). Among Lrp orthologs, unique residues classify them for their local vs. global function relevance (Unoarumhi et al., 2016), which is yet to be explored in many Gram-positive bacteria. Considering all features, we hypothesized that *C. difficile* Lrp may exhibit similar functional features to those of *E. coli* Lrp as a global regulator.

To understand the transcriptional status of *lrp* in *C. difficile*, we analyzed *lrp* expression under consideration of lag phase (2 h) as basal expression. The growth phase–specific *lrp* expression in cells of *C. difficile* strains 630Δ*erm* and R20291 cells showed the highest relative expression at the mid-log phase time point (8 h; Figure 2A). A distinct *lrp* expression profile in strain R20291 showed longer retention up to the late log phase compared with the basal level (2 h; Figure 2A); by contrast, strain 630Δ*erm* exhibited lower expression in the late log phase compared to mid-log (Figure 2B). Whether the differences in the *lrp* expression patterns of strains R20291 and 630Δ*erm* were significant was unclear; however, the observed increased in *lrp* expression during logarithmic growth was in accordance with corresponding observations from another study (Hung et al., 2015).

Beloin et al. (1997) demonstrated that in *B. subtilis, lrp* mutation led to the transitory inhibition of growth in a minimal medium in the presence of only valine and isoleucine; this inhibition was relieved by leucine. The present study showed that in a rich medium, the growth curves of the parental strains, *lrp* mutants, and complemented strains exhibited no drastic differences in terms of growth kinetics (Figures 3A,B). However, in the minimal medium (CDMM), the *lrp* mutants showed a marginal delay in the onset of the logarithmic phase but reached similar final optical density at 15 h (Figures 3C,D). In *E. coli*, the growth of the *lrp* mutant in a glucose minimal medium is significantly slower than that of wild type strains; however, this growth defect can be restored by the addition of L-serine and L-leucine (Ambartsoumian et al., 1994; Newman and Lin, 1995). Interestingly, in the case of the *C. difficile lrp* mutants, the addition of either or both amino acids did not restore growth, even under nutrient limiting conditions (data not shown). Nevertheless, our results demonstrated that *C. difficile lrp* plays a role in growth phase transition. In future experiments, we intend to address the specific role, if any, that *C. difficile lrp* plays in amino acid metabolism.

In Gram-positive bacteria, most experiments on the function of *lrp* are performed using non-pathogenic bacteria, *lrp* is generally not regarded as a virulence regulator. It has been known that many virulence factors of a pathogen are co-regulated depending on the nutritional state of the bacteria. Because the primary determinant of pathogenicity in *C. difficile* is the production of toxins A and B, we hypothesized that Lrp may play a role in toxin production. Both protein and gene expression analysis demonstrated that Lrp is a repressor of toxin A and B production, and this repression occurs at the transcriptional level. Furthermore, the increased level of toxin production through inactivation of *lrp* led to enhanced cytotoxicity against cultured cells and an overall increase in *C. difficile*–associated diarrhea and inflammation in animal infection studies.

Because the *lrp* mutants exhibited notably high toxin A and B levels and multiple fold increases in cytotoxicity, we deciphered the expression levels of genes known to encode for regulators of toxin production. Our results indicated that the regulation of toxin A and B expression by Lrp is primarily enabled by the repression of positive regulators TcdR. In addition, Lrp appeared to influence the expression of other known toxin regulators in a strain-specific manner. In R20291, the deletion of *lrp* resulted in significantly higher expression of TcdC, whereas no such differences were observed in the expression of TcdC in strain 630Δerm. However, because the role of TcdC as a toxin gene regulator remains under debate, whether the increased expression of *tcdC* in the R20291 *lrp* mutant strain plays a role in regulating toxin production remains unclear. Furthermore, Lrp in R20291 affected *codY* transcription except for in strain 630Δerm, whereas the opposite held true for *ccpA* expression. Because numerous reports have shown that the regulation of toxin production is multi-factorial and in some cases ribotype specific, the regulation of toxin expression by Lrp is likely highly complicated (Stabler et al., 2009; Mackin et al., 2013; Girinathan et al., 2017; Daou et al., 2019). Gaining further understanding of toxin regulation by Lrp would involve further transcriptomic and ChIP-Seq studies. Furthermore, representatives from multiple ribotypes would need to be evaluated together to elucidate the complexity of the system.

The strain-specific regulation of virulence traits in *C. difficile* appears to extend to sporulation. One study reported that in *B. subtilis*, Lrp may play a role in entering the sporulation phase, either by controlling the factors that trigger the onset of sporulation or regulating early sporulation genes (Beloin et al., 1997). Further, it also demonstrated earlier onset of sporogenesis in the *lrp* mutant than in the reference strain, suggesting that the *B. subtilis* Lrp protein plays a role in the growth phase transition (Beloin et al., 1997). The present study analyzed the sporulation efficiency for both backgrounds involving their parental strains, *lrp* mutants, and complemented strains. In R20291, *lrp* mutation appeared to repress sporulation (Figure 7A). By contrast, the *lrp* mutation in strain 630Δerm exhibited no statistically significant differences in sporulation frequency (Figure 7B). The repressive effect of *lrp* on sporulation in R20291 is due partly to the repression of the master regulator spo0A. Interestingly, although no phenotypical differences were observed in the sporulation efficiency of the 630 *lrp* mutant strain, the expression level of spo0A was significantly downregulated. Further, we also studied the expression levels of co-expressed genes during sporulation (e.g., *sig*E, *sig*F, *sig*G, and CD1476/CD1579). A previous transcriptome study indicated the repression of spore-associated genes (*sig*E, *sig*F, *sig*G, and *sig*K), leading to a reduction in the sporulation rate and the quantity of heat-resistant spores (Girinathan et al., 2017). The present study observed that all the mentioned genes except for CD1476 were highly expressed in the R20291 *lrp* mutant strain (Supplementary Figure 2), and this observation strongly corroborated our findings on sporulation efficiency and *spo0A* expression level. However, *lrp* mutation in strain 630Δerm exhibited the opposite effect (Supplementary Figure 2). It is possible that in strain 630, the regulation of these sporulation genes by Lrp may not have significantly altered the sporulation rate; however, more detailed analysis is required to understand this phenomenon.

According to Antunes et al. (2012), the glucose-activated CcpA protein is a negative regulator of both the *tcd* gene cluster and the *spo0A* and *sigF* genes in ribotype 027. In strain R20291, TcdR is a positive regulator of sporulation as well as toxin synthesis (Girinathan et al., 2017). In strain 630, the RstA protein has been reported as an activator of sporulation but an inhibitor of toxin synthesis (Edwards et al., 2016), and in the same strain another study demonstrated that a *spo0A* mutation caused overexpression of the *tcdA* gene (Pettit et al., 2014); this finding contradicted that of an *in vivo* study that showed no significant effects of *spo0A* mutation on toxin production (Rosenbusch et al., 2012). A subsequent study involving a ribotype 027 strain detected over-expression of both *tcdA* and *tcdB* in a *spo0A* mutant; however, no transcriptional effects were observed for the *spo0A* mutant in strain 630 (Mackin et al., 2013). Further evidence of the strain-specific regulation of virulence traits by *lrp* is provided by findings related to swimming motility. In our study, *lrp* appeared to affect motility in R20291 but not in 630Δerm (Supplementary Figures 3A,B), whereas biofilm formation was not an *lrp*-regulated trait (Supplementary Figure 5). The decrease in motility exhibited by the R20291 *lrp* mutant might be explained by a decrease in the transcriptional level of the flagellar regulator *sigD*. No change was observed in *sigD* transcriptional level in 630Δerm *lrp* which also correlated with the observed unchanged motility phenotype. Recently Anjuwon-Foster et al. described a complex regulation and phase variable orientation of the early stage flagellar operon in *C. difficile*. The orientation of the flagellar switch determines multiple flagellar gene expression, including *sigD* (Anjuwon-Foster et al., 2018). Phase on (flg-on state) led to flagellum production, swimming motility, and high toxin production. Further, the phase-variable production of flagella and toxins was thought to balance the benefits of swimming motility and toxin production during the course of infection. SigD is one of the many players involved in controlling toxin production, and has been shown to be a positive regulator via direct binding to the promoter region of tcdR (El Meouche et al., 2013). In our study, Lrp appears to be a positive regulator of sigD in R20291. However, Lrp also have been shown in this study to be a repressor of toxin production via transcriptional regulation of multiple toxin regulators. Although much information regarding the extent of the regulation exerted by Lrp on *C. difficile* virulence traits remains unknown, it is clear that such regulation is likely strain-specific, perhaps even ribotype-specific, and involves many more factors that can only be resolved by extensive genome-wide analysis.

Finally, the significance of Lrp as a virulence factor was demonstrated by the mouse model of infection. The severity of infection was clearly indicated in the mice infected with the R20291 *lrp* mutant strain, attributed to smaller cecum size and less well-formed feces, as well as more extensive necrosis and inflammation, as revealed by histological examination. It has been suggested in the past that clindamycin administration prior to challenge with *C. difficile* select for Clostron-based mutants bearing the *ermB* cassette, although other studies in which Clostron-based mutants with reduced virulence *in vivo* have also been reported (Ünal et al., 2018; Zhu et al., 2019). However, for further clarification, we are in the process of obtaining a markerless *lrp* mutant using the recently developed RiboCas system (Cañadas et al., 2019). To further reveal the role of Lrp in *C. difficile* pathogenesis, we will perform animal studies that will include measurement of bacterial burden in feces and cecum, and compare the colon inflammatory cytokine response between wild type and *lrp* mutants in diverse *C. difficile* strains to clarify any unique mode of strain-specific infection progression.

Taken together, this is the first report detailing a functional analysis of *lrp* in a Gram-positive pathogen. *C. difficile* Lrp is involved in growth phase transition. Notably, *C. difficile* Lrp was demonstrated as a novel virulence regulator involved in toxin production, sporulation, and swimming motility. Further study on the role of Lrp in other Gram-positive pathogens and whether it plays a role in regulating pathogenicity is warranted. Future studies will aim to unravel the extent of the Lrp-regulon and how it contributes to the diverse regulation of virulence traits in *C. diffiicile*.

## Data Availability Statement

All datasets generated for this study are included in the manuscript/Supplementary Files.

## Ethics Statement

The animal study was reviewed and approved by IACUC of NCKU (approval no. NCKU-IACUC-102-149).

## Author Contributions

I-HH and J-WC designed the experiments. K-YC, JR, and Y-CC carried out the experiments. K-YC, JR, Y-CC, J-WC, P-JT, and I-HH analyzed the data. K-YC, JR, and I-HH wrote the manuscript.

### Conflict of Interest

The authors declare that the research was conducted in the absence of any commercial or financial relationships that could be construed as a potential conflict of interest.

## Supplementary Material

Supplementary Figure 1 Multiple sequence alignment showing identical Lrp aminoacid sequences of *C. difficile* strains CD196, R20291, 630, 630Δerm, and R1 (^*^conserved residues).

Supplementary Figure 2 Lrp affects sporulation-related transcriptional factors in a strain-specific manner. The transcriptional levels of sporulation-associated transcriptional factors *sigE, sigF, sigG*, and CD1476 (R20291)/CD1579 (630Δerm) were analyzed using qRT-PCR from culture grown to the late exponential to early stationary phase in SMC medium. In the case of the R20291 *lrp* mutant strain, significantly higher transcriptional levels were observed for *sigE, sigF, and sigG* except CD1476; by contrast, all the representative genes exhibited down-regulation for the *lrp* mutant strain of 630Δerm; 16s ribosomal RNA was used for reference. Data are represented as the mean ± standard error of the mean, and the results are representative of at least three independent experiments (ns, not significant; ^*^*p* < 0.01; ^***^*p* < 0.001, ^****^*p* < 0.0001).

Supplementary Figure 3 Lrp affects motility in strain R20291 but not in strain 630Δerm. Motility was assessed by stab inoculation and extent of motility was visualized 24 h post inoculation by photograph (left panel). The *fliC* mutant served as the negative control. **(A)** R20291 and **(B)** 630Δerm.

Supplementary Figure 4 Inactivation of *lrp* showed a strain-specific transcriptional regulation of *sigD* (a known transcriptional regulator of motility) in strain R20191 and 630Δerm. 16s ribosomal RNA was used for reference. Data are represented as the mean ± standard error of the mean, and the results are representative of at least three independent experiments [WT, wild type (parental strain); ns, not significant. ^****^*p* < 0.0001].

Supplementary Figure 5 Lrp does not affect biofilm formation in both *C. difficile* R20291 and 630Δerm. Twenty four hours biofilm was measured by crystal violet staining. Methanol-extracted dye was quantified by measuring absorbance at 595 nm. A comparison between the parental strain and its *lrp* mutant along with the complemented strain was conducted. Data were analyzed by one-way analysis of variance and Dunnett's multiple-comparison test. **(A)** R20291; **(B)** 630Δerm [WT, wild type (parental strain); ns, not significant].
