# Supplementary figures and images for: The Transcriptional Regulator Lrp Contributes to Toxin Expression, Sporulation, and Swimming Motility in Clostridium difficile
Source: Front Cell Infect Microbiol. 2019 Oct 17;9:356. doi: 10.3389/fcimb.2019.00356 (PMC6811523; doi:10.3389/fcimb.2019.00356)

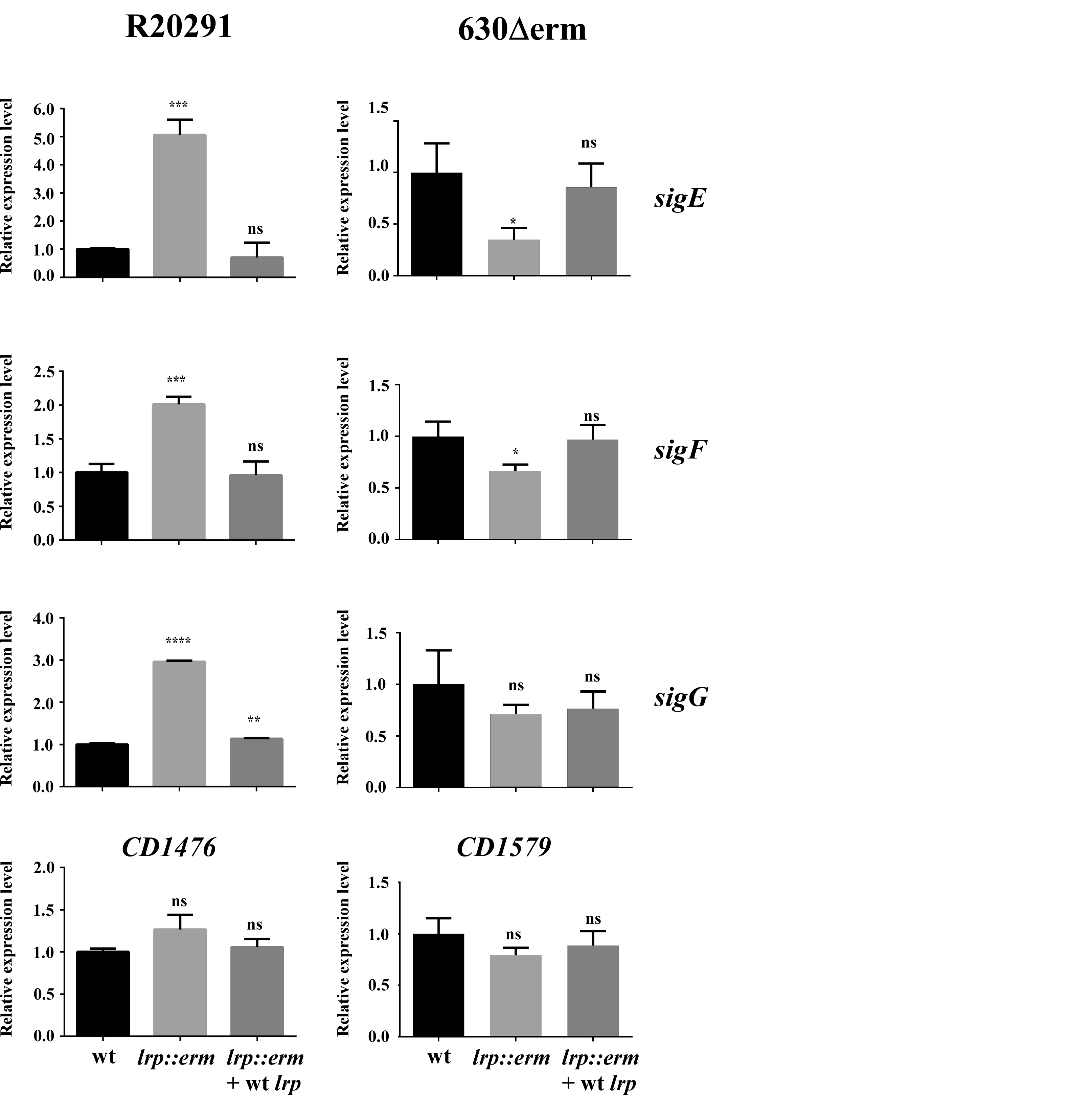

Supplement: Supplementary Figure 2 — Lrp affects sporulation-related transcriptional factors in a strain-specific manner. The transcriptional levels of sporulation-associated transcriptional factors sigE, sigF, sigG, and CD1476 (R20291)/CD1579 (630Δerm) were analyzed using qRT-PCR from culture grown to the late exponential to early stationary phase in SMC medium. In the case of the R20291 lrp mutant strain, significantly higher transcriptional levels were observed for sigE, sigF, and sigG except CD1476; by contrast, all the representative genes exhibited down-regulation for the lrp mutant strain of 630Δerm; 16s ribosomal RNA was used for reference. Data are represented as the mean ± standard error of the mean, and the results are representative of at least three independent experiments (ns, not significant; *p < 0.01; ***p < 0.001, ****p < 0.0001). [file Image_2.TIF]

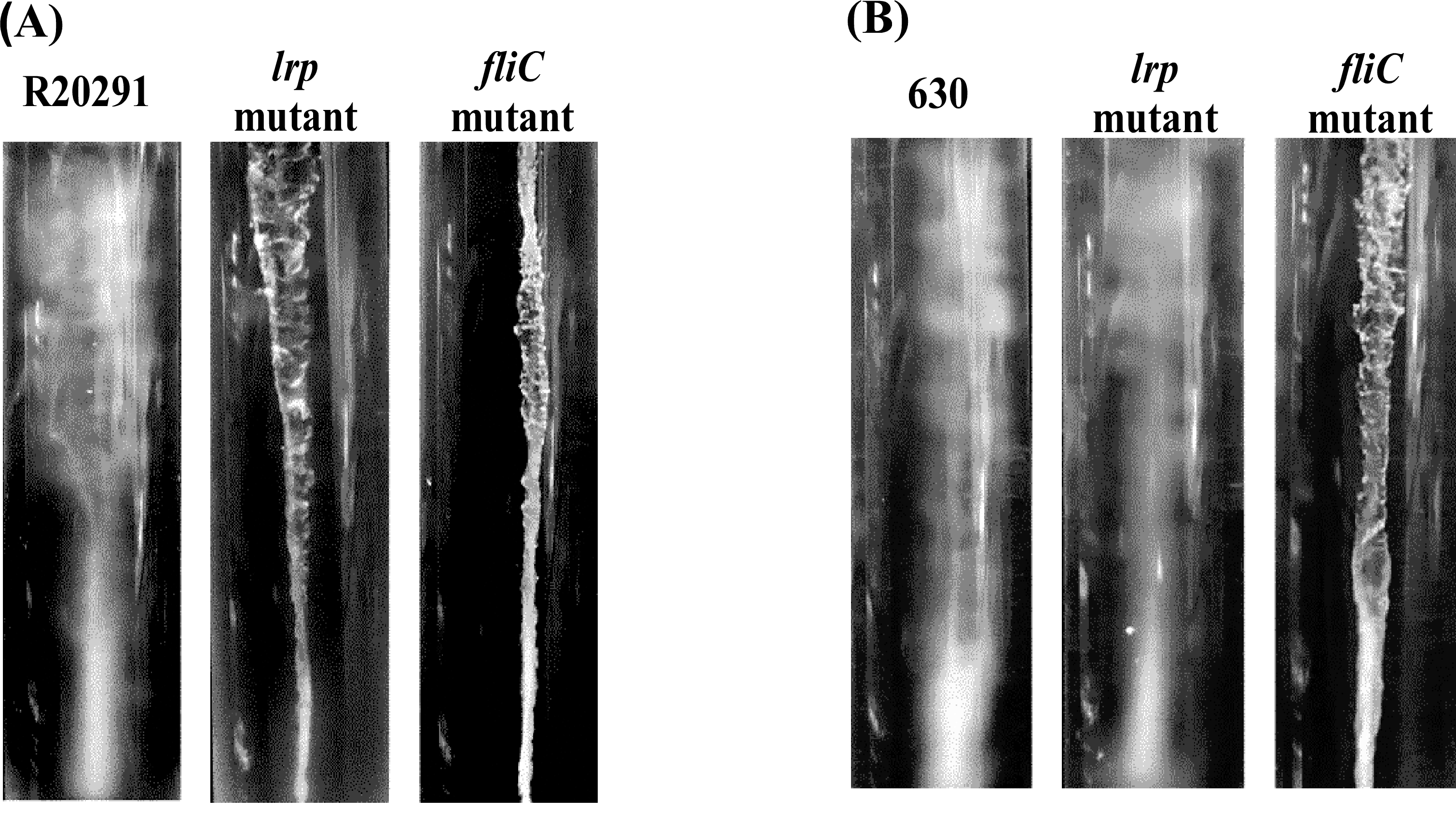

Supplement: Supplementary Figure 3 — Lrp affects motility in strain R20291 but not in strain 630Δerm. Motility was assessed by stab inoculation and extent of motility was visualized 24 h post inoculation by photograph (left panel). The fliC mutant served as the negative control. (A) R20291 and (B) 630Δerm. [file Image_3.TIF]

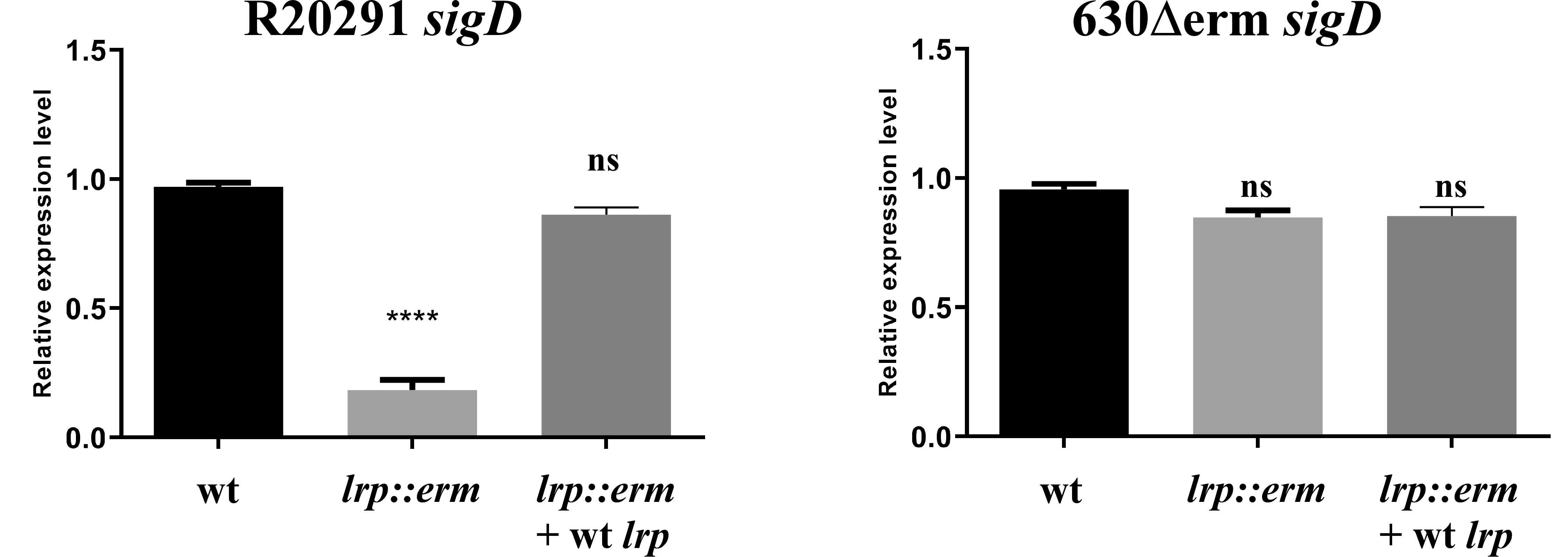

Supplement: Supplementary Figure 4 — Inactivation of lrp showed a strain-specific transcriptional regulation of sigD (a known transcriptional regulator of motility) in strain R20191 and 630Δerm. 16s ribosomal RNA was used for reference. Data are represented as the mean ± standard error of the mean, and the results are representative of at least three independent experiments [WT, wild type (parental strain); ns, not significant. ****p < 0.0001]. [file Image_4.TIF]

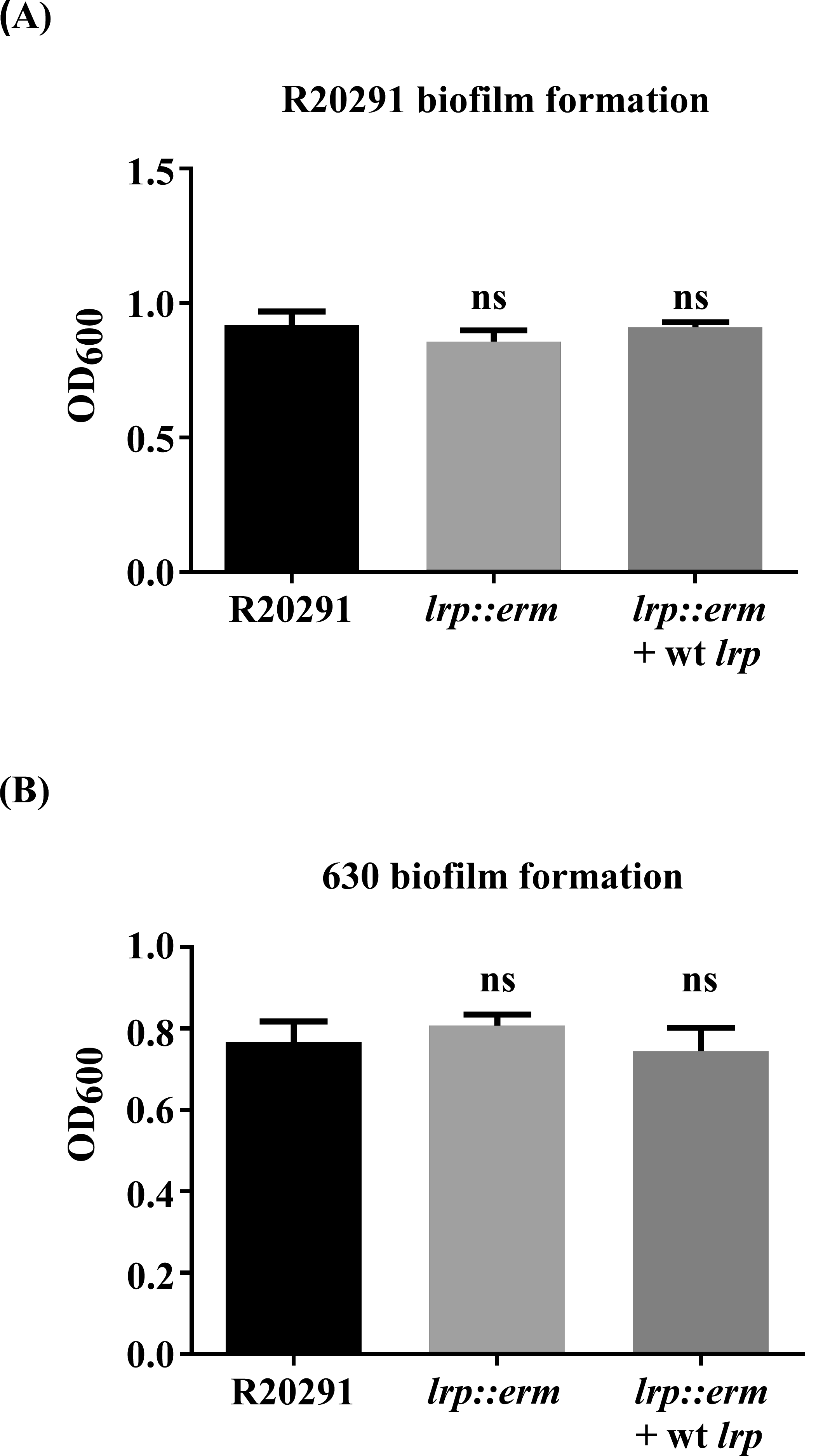

Supplement: Supplementary Figure 5 — Lrp does not affect biofilm formation in both C. difficile R20291 and 630Δerm. Twenty four hours biofilm was measured by crystal violet staining. Methanol-extracted dye was quantified by measuring absorbance at 595 nm. A comparison between the parental strain and its lrp mutant along with the complemented strain was conducted. Data were analyzed by one-way analysis of variance and Dunnett's multiple-comparison test. (A) R20291; (B) 630Δerm [WT, wild type (parental strain); ns, not significant]. [file Image_5.TIF]
